# Comorbidities and COVID-19 status influence the survival rate of geriatric patients in intensive care units: a prospective cohort study from the Indonesian Society of Anaesthesiology and Intensive Therapy

**DOI:** 10.1186/s12877-022-03227-9

**Published:** 2022-06-25

**Authors:** Nancy Margarita Rehatta, Susilo Chandra, Djayanti Sari, Mayang Indah Lestari, Tjokorda Gde Agung Senapathi, Haizah Nurdin, Belindo Wirabuana, Bintang Pramodana, Adinda Putra Pradhana, Isngadi Isngadi, Novita Anggraeni, Kenanga Marwan Sikumbang, Radian Ahmad Halimi, Zafrullah Khany Jasa, Akhyar Hamonangan Nasution, Mochamat Mochamat, Purwoko Purwoko

**Affiliations:** 1 Department of Anaesthesiology and Reanimation, Faculty of Medicine, Universitas Airlangga, Surabaya, Indonesia; 2 Department of Anaesthesiology and Intensive Care, Faculty of Medicine, Universitas Indonesia, Jakarta, Indonesia; 3 Department of Anaesthesiology and Intensive Therapy, Faculty of Medicine, Public Health, and Nursing, Universitas Gadjah Mada, Yogyakarta, Indonesia; 4 Department of Anaesthesiology and Intensive Therapy, Faculty of Medicine Universitas Sriwijaya, Jenderal Sudirman Street KM. 3.5, Palembang, South Sumatera 30126 Indonesia; 5 Department of Anaesthesiology and Intensive Therapy, Faculty of Medicine, Universitas Udayana, Denpasar, Indonesia; 6 Department of Anaesthesiology and Intensive Care, Faculty of Medicine, Universitas Hasanuddin, Makassar, Indonesia; 7 Department of Anaesthesiology and Intensive Therapy, Faculty of Medicine, Universitas Brawijaya, Malang, Indonesia; 8 Department of Anaesthesiology and Intensive Therapy, Faculty of Medicine, Universitas Riau, Pekanbaru, Riau Indonesia; 9 Department of Anaesthesiology and Intensive Care, Faculty of Medicine, Universitas Lambung Mangkurat, Banjarmasin, Indonesia; 10 Department of Anaesthesiology and Intensive Care, Faculty of Medicine, Universitas Padjadjaran, Bandung, Indonesia; 11 Department of Anaesthesiology and Intensive Therapy, Faculty of Medicine, Universitas Syiah Kuala, Banda Aceh, Indonesia; 12 Department of Anaesthesiology and Intensive Therapy, Faculty of Medicine, Universitas Sumatera Utara, Medan, Indonesia; 13 Department of Anaesthesiology and Intensive Therapy, Faculty of Medicine, Universitas Diponegoro, Semarang, Indonesia; 14 Department of Anaesthesiology and Intensive Therapy, Faculty of Medicine, Universitas Sebelas Maret, Surakarta, Indonesia

**Keywords:** Intensive care, Geriatric, Characteristics, Outcome

## Abstract

**Background:**

With the more advanced science in the field of medicine and disease management, the population of geriatric intensive care patients is increasing. The COVID-19 pandemic has impacted healthcare management around the globe, especially on critically-ill elderly patients. We aim to analyse the relationship between underlying illnesses, including COVID-19, and the survival rate of elderly patients who are treated in the intensive care setting.

**Methods:**

We conducted a prospective cohort study at 14 teaching hospitals for Anaesthesiology and Intensive Therapy Education in Indonesia. We selected all subjects with 60 years of age or older in the period between February to May 2021. Variables recorded included subject characteristics, comorbidities, and COVID-19 status. Subjects were followed for 30-day mortality as an outcome. We analysed the data using Kaplan-Meier survival analysis.

**Results:**

We recruited 982 elderly patients, and 728 subjects were in the final analysis (60.7% male; 68.0 ± 6.6 years old). The 30-day mortality was 38.6%. The top five comorbidities are hypertension (21.1%), diabetes (16.2%), moderate or severe renal disease (10.6%), congestive heart failure (9.2%), and cerebrovascular disease (9.1%). Subjects with Charlson’s Comorbidity Index Score > 5 experienced 66% death. Subjects with COVID-19 who died were 57.4%. Subjects with comorbidities and COVID-19 had lower survival time than subjects without those conditions (*p* < 0.005). Based on linear correlation analysis, the more comorbidities the geriatric patients in the ICU had, the higher chance of mortality in 30 days (*p <* 0.005, R coefficient 0.22).

**Conclusion:**

Approximately one in four elderly intensive care patients die, and the number is increasing with comorbidities and COVID-19 status.

## Introduction

Each year, medical technology develops tremendously as diseases management becomes more effective and comprehensive. Unfortunately, this revelation could appear as a double-edged sword to the world of medicine. The bright side is that many people will exceed typical life expectancy, not to mention there is also a trend of decreasing mortality. Consequently, countries face a tremendous burden because more older people will require general and specialist healthcare services. According to a global survey by WHO, more than 700 million people are older than 65 years in 2020 and will continue to double by 2050 [1]. Indonesia itself has been the epicentre of older persons in Southeast Asia, with a survey revealing that almost 10% of its population were elderly [2].

Aging is an inevitable fate. With age, human physiological functions are diminished, making them more prone to illness [3]. The so-called geriatric comorbidities appear, such as reduced cognitive function, frailty, elevated mean arterial pressure, decreased oxyhaemoglobin in blood, decreased glomerular filtration rate, and many more [1]. These conditions cause elderly patients to succumb to complications and require good care in intensive care units (ICU). Therefore, the elderly patients’ domination of the ICU population is understandable. In 2017, a global multi-centred survey showed that the average age of patients admitted to ICU was 60 years old [4]. Several studies, such as in Canada and Europe, claimed that the ICU admission rate rose exponentially by the time patients reached the age of 40. The rate will reach its peak in the population of 80. In addition, patients older than 70 years have twice the risk of being admitted to the ICU than other age groups [5, 6]. These unfortunate situations are correlated with the increase of morbidity and mortality in the elderly, accompanied by increasing medical costs. The ICU took up 8% of the allocation of health care funds in Indonesia in 2011 [7].

The COVID-19 global pandemic has had an impact on every demographic, especially the elderly. COVID-19 has become one of the conditions that affect the survivability of elderly patients admitted to hospitals as it can hinder the already immunocompromised status of older people [8]. Indonesian COVID-19 Task Force data confirmed that as many as 53% of patients with COVID-19 admitted to the ICU were elderly patients with the age group older than 60 years had the lowest recovery rate (9.4%) and the highest mortality (41%) compared to other groups [9]. Thus, in planning good clinical management of this population, intensivists need to apprehend the clinical characteristics and prognosticate the geriatric patients admitted to the ICU. In developing countries, there is still a lack of sufficient studies that determine both prevalence and survivability in geriatric patients admitted to ICU. This study aims to describe the characteristics and analyse the survival rate in elderly patients treated in the ICU in Indonesia, especially those with comorbidities and COVID-19.

## Methods

### Study design

Our study design was a cohort study. We researched 14 tertiary teaching hospitals that also act as teaching hospitals for Anaesthesiology and Intensive Therapy Residency Education in Indonesia. We briefed and standardized each research team in respective before the data collection. We carried out the data collection for 3 months, from February to May 2021.

### Participants

We analysed patients who were admitted to the ICU with both COVID-19 and non-COVID-19 patients. We included all ICU patients aged 60 years or older in the period between February to May 2021, using whole sampling method. We excluded all subjects that failed to be followed within the observation period such as incomplete medical record data.

### Ethical clearance

We received our ethical approval from institutional review boards of Universitas Gadjah Mada Faculty of Medicine’s Medical and Health Research Ethics Committee (MHREC) - Dr. Sardjito General Hospital with registry number KE/FK/1381/EC/2020.

### Geriatric parameters

The recorded variables were subject characteristics, comorbidities, and COVID-19 status. Subject characteristics included age, gender, body mass index, comorbidities based on Charlson’s Comorbidity Index Score, MSOFA score, underlying cause of admission, ventilator use, inotropic use, vasopressor use, COVID-19 status, duration of mechanical ventilation use, and the length of ICU stay. Charlson’s Comorbidity Index Score is classified to four levels of groups based on the risk [10]. The underlying cause of admission is to two causes; first cause is surgical which is all underlying causes of ICU admission related to the postoperative indication such as stabilization, and second cause is medical which is all underlying causes of ICU admission that are not related to post-operative indications. The MSOFA score consists of six parameters, including the respiratory system as assessed by the ratio of PaO_2_ and FiO_2_, the hepatic system as assessed by bilirubin, the cardiovascular system based on the mean arterial pressure, coagulation function based on the platelet value, the central nervous system based on the Glasgow Coma Scale, and the renal system as assessed by serum creatinine [11]. We established the diagnosis of COVID by examining patients’ presence of the nucleic acid of SARS-Cov-2 using a real-time polymerase chain reaction machine.

### Outcome measure

The output of this study is mortality within 30 days after being recruited in the ICU. Subjects who could not be followed up were dropped out from the study.

### Statistical analysis

We conducted the statistical analysis using the SPSS software program. Numerical variables with normal distribution are presented as mean ± deviation or otherwise in the form of a median (minimum-maximum), whereas percentage represents categorical variables. We deliver the data in the form of tables and narratives. After that, we used Kaplan-Meier survival analysis to describe the survival rate invariable that we tested. If *p* < 0.05, we display it in figure form.

## Results

**Fig. 1:**
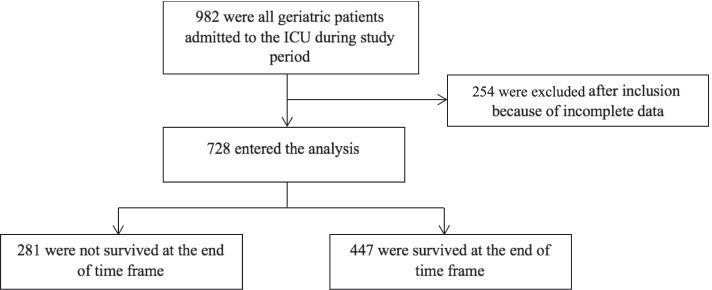
Study Flowchart

We recruited 982 subjects, of which we excluded 254 subjects due to incomplete data, so 728 were analysed (Fig. 1). Table 1 presents the characteristics of all the recruited subjects. The average age of the subject 68.0 ± 6.6 years and most of them are male. Subjects on average had an ideal body mass index and more than 50 % were admitted for medical reasons apart from surgical (Table 1). The mean MSOFA score at admission was 5.1 ± 3.7 and decreased to a mean of 4.8 ± 5.8 30 days later. Approximately two out of three subjects admitted to the ICU required ventilator support (62.4%) and more than a third of the subjects required vasopressor support (38.2%). The average length of ICU stay was 6.9 ± 7.0 days, with the length of use of the ventilator being 4.0 ± 6.4 days. The 30-day mortality was among 281 geriatrics (38.6%) with ICU admission, whereas 447 of them (61.4%) survived. Subjects with Charlson’s Comorbidity Index Score greater than 5 experienced deaths more often with 66%. Elderly people in the ICU with COVID-19 who died were 56.4% among 289 subjects, and bivariate analysis shows that COVID-19 status causes 17.62 times greater risk of dying in elderly patients admitted to the ICU. About 77.2% of all subjects presented with at least one comorbidity, and the presence of at least one comorbidity cause 3.5 times greater risk of dying by the end of 30 days in geriatrics admitted to the ICU. The mSOFA on the day of admission and at the end of the observation were both statistically higher in non-survivors compared to survivors. Non-survivors had statistically significant shorter duration of length of stay and duration of mechanical ventilation compared to the survivors.

**Table 1:** Subject Characteristics

Characteristics	Survivors *n =* 447 (61.4%)	Non-Survivors *n =* 281 (38.6%)	Total *n =* 728 (100%)	*p*-value (OR; CI 95%)
Age, years old	67.3 ± 6.4	69.2 ± 6.9	68.0 ± 6.6	< 0.005**
Gender, n (%)				0.99
Male	275 (62.2%)	167 (37.8%)	442 (60.7%)	
Female	178 (62.2%)	108 (37.8%)	286 (29.3%)	
Body Mass Index, kg/m^2^	23.1 ± 7.6	23.9 ± 3.8	23.9 ± 6.2	0.063
Weight, kg	61.8 ± 11.8	62.8 ± 10.6	62.1 ± 11.3	
Height, cm	160.6 ± 10.1	161.7 ± 6.5	161.0 ± 8.7	
Charlson’s Index Score, n (%)				< 0.005*
Score 0	180 (74.4%)	62 (25.6%)	242 (33.2%)	
Score 1-2	211 (61.5%)	132 (38.5%)	343 (47.1%)	
Score 3-4	45 (48.4%)	48 (51.6%)	93 (12.8%)	
Score > 5	17 (34%)	33 (66%)	50 (6.9%)	
Presence of Comorbidities				< 0.005* (3.5; 2.28-5.38)
No comorbidity	136 (81.9%)	30 (18.1) %	166 (22.8%)	
At least one	317 (56.4%)	245 (43.6%)	563 (77.2%)	
Diagnosis, n (%)				< 0.005* (3.43; 2.46-4.78)
Surgical	238 (78%)	67 (22%)	305 (41.9%)	
Medical	215 (50.8%)	208 (49.2%)	423 (58.1%)	
Use of Ventilators, n (%)				< 0.005* (10.48; 6.80-16.15)
No	246 (89.8%)	28 (10.2%)	274 (37.6%)	
Yes	207 (45.6%)	247 (54.4%)	454 (62.4%)	
Use of Inotropic, n (%)				< 0.005* (12.40; 8.51-18.07)
No	400 (79.4%)	104 (20.6%)	504 (69.2%)	
Yes	53 (23.7%)	171 (76.3%)	224 (30.8%)	
Use of Vasopressor, n (%)				< 0.005* (3.5; 2.28-5.381)
No	384 (85.3%)	66 (14.7%)	405 (55.6%)	
Yes	69 (24.8%)	209 (75.2%)	278 (38.2%)	
COVID-19 Status, n (%)				< 0.005* (17.62; 12.08-25.69)
Non-COVID	327 (74.5%)	112 (25.5%)	439 (60.3%)	
COVID-19	126 (43.6%)	163 (56.4%)	289 (39.7%)	
MSOFA Score initial	3.8 ± 2.9	7.0 ± 3.8	5.1 ± 3.7	< 0.005**
MSOFA Score Day-30	1.8 ± 2.3	9.2 ± 6.5	4.8 ± 5.8	< 0.005**
Length of stay in ICU, days	5.9 ± 6.5	8.2 ± 7.6	6.9 ± 7.0	< 0.005**
Duration of mechanical ventilation, days	2.4 ± 5.3	6.3 ± 7.1	4.0 ± 6.4	< 0.005**

*Chi-square test; **Mann-Whitney test

According to Table 2, the most common comorbidity was hypertension; which was found in about one in five subjects (21.1%). Comorbidities with significant difference between survivors and non-survivors are hypertension (*p*-value 0**.**002**;** OR 1.65 [CI 95% 1.19-2.30]), diabetes with (p-value < 0.005**;** OR 5.83 [CI 95% 2.31-14.73]) and without complications (*p*-value 0.002**;** OR 1.73 [CI 95% 1.21-2.47]), moderate-to-severe renal disease (*p*-value < 0.005**;** OR 2.77 [CI 95% 1.81-4.23]), congestive heart failure (p-value 0.046**;** OR 1.56 [CI 95% 1.0-2.43]), cerebrovascular disease (p-value 0.005**;** OR 1.86 [CI 95% 1.19-2.91]), and chronic pulmonary disease (*p*-value 0.031**;** OR 2.27 [CI 95% 1.05-4.87]). Table 3 presents positive yet weak correlation between Charlson Index on admission day to the mortality chance on the 30th day (p-value < 0.005; R coefficient 0.22) and mSOFA score at the end of ICU stay (p-value < 0.005; R coefficient 0.27), whereas there is a positive and very weak correlation between Charlson Index on admission day to the duration of ventilation support use (p-value < 0.005; R coefficient 0.13).

**Table 2:** Comorbidities

Comorbidities	Survivors *n =* 317 (56.4%)	Non-Survivors *n =* 245 (43.6%)	Total (*n =* 563)	p-value (OR; CI 95%)
Hypertension	112 (53.6%)	97 (46.4%)	209 (21.1%)	0.002* (1.65; 1.19-2.30)
Diabetes	83 (51.9%)	77 (48.1%)	160 (16.2%)	0.002* (1.73; 1.21-2.47)
Moderate-to-severe renal disease	43 (41%)	62 (59%)	105 (10.6%)	< 0.005* (2.77; 1.81-4.23)
Congestive heart failure	48 (52.7%)	43 (47.3%)	91 (9.2%)	0.046* (1.56; 1.0-2.43)
Cerebrovascular disease	44 (48.9%)	46 (51.1%)	90 (9.1%)	0.005* (1.86; 1.19-2.91)
Tumour (benign or malignant)	56 (62.9%)	33 (37.1%)	89 (9.0%)	0.885
Prior myocardial infarction	41 (70.7%)	17 (29.3%)	58 (5.9%)	0.166
Peripheral vascular disease	24 (57.1%)	18 (42.9%)	42 (4.2%)	0.484
Chronic pulmonary disease	12 (42.9%)	16 (57.1%)	28 (2.8%)	0.031* (2.27; 1.05-4.87)
Diabetes with chronic complications	6 (23.1%)	20 (76.9%)	26 (2.6)	< 0.005* (5.83; 2.31-14.73)
Moderate or severe liver disease	10 (43.5%)	13 (56.5%)	23 (2.3%)	0.06
Mild liver disease	11 (53.4%)	10 (47.6%)	21 (2.1%)	0.345
Dementia	5 (38.5%)	8 (61.5%)	13 (1.3%)	0.075
Non-CVD neurologic disease	7 (53.8%)	6 (46.2%)	13 (1.3%)	0.529
Metastatic solid tumor	6 (50%)	6 (50%)	12 (1.2%)	0.378
Peptic ulcer disease	3 (50%)	3 (50%)	6 (0.6%)	0.535
Rheumatologic disease	0	2 (100%)	2 (0.2%)	0.069
Leukemia	0	1 (100%)	1 (0.1%)	0.199
Lymphoma	0	0	0 (0.0%)	n/a
Acquired immunodeficiency syndrome (AIDS)	0	0	0 (0.0%)	n/a

*Chi-square test

**Table 3:** Correlation analysis

Charlson Index at admission	R coefficient	p-value
Mortality in 30 days	0.22	< 0.005*
mSOFA at the end of ICU stay	0.27	< 0.005*
Duration of ventilation support use in the ICU	0.13	< 0.005*

*Spearman correlation test

**Fig. 2:**
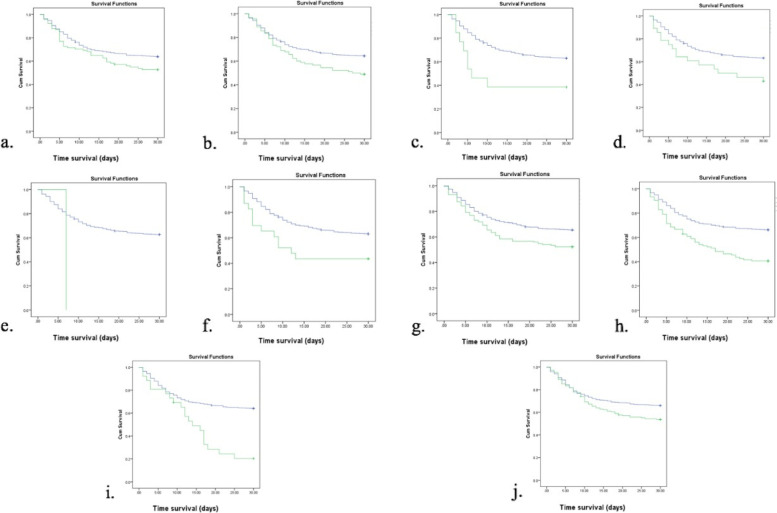
Comorbidities and survivability. Blueline is without comorbid, and the green line is with comorbid. (a) congestive heart failure (*p =* 0.043); b. cerebrovascular disease (log-rank *p =* 0.008); c. dementia (log-rank *p =* 0.015); d. chronic pulmonary disease (log-rank *p =* 0.02); e. rheumatologic disease (log-rank *p =* 0.005); f. moderate to severe liver disease (log-rank *p =* 0.015); g. diabetes (log-rank *p =* 0.002); h. moderate-to-severe renal disease (log-rank *p =* 0.001); i. diabetes with chronic complications (log-rank *p =* 0.001); j. hypertension (log-rank *p =* 0.005)

**Fig. 3:**
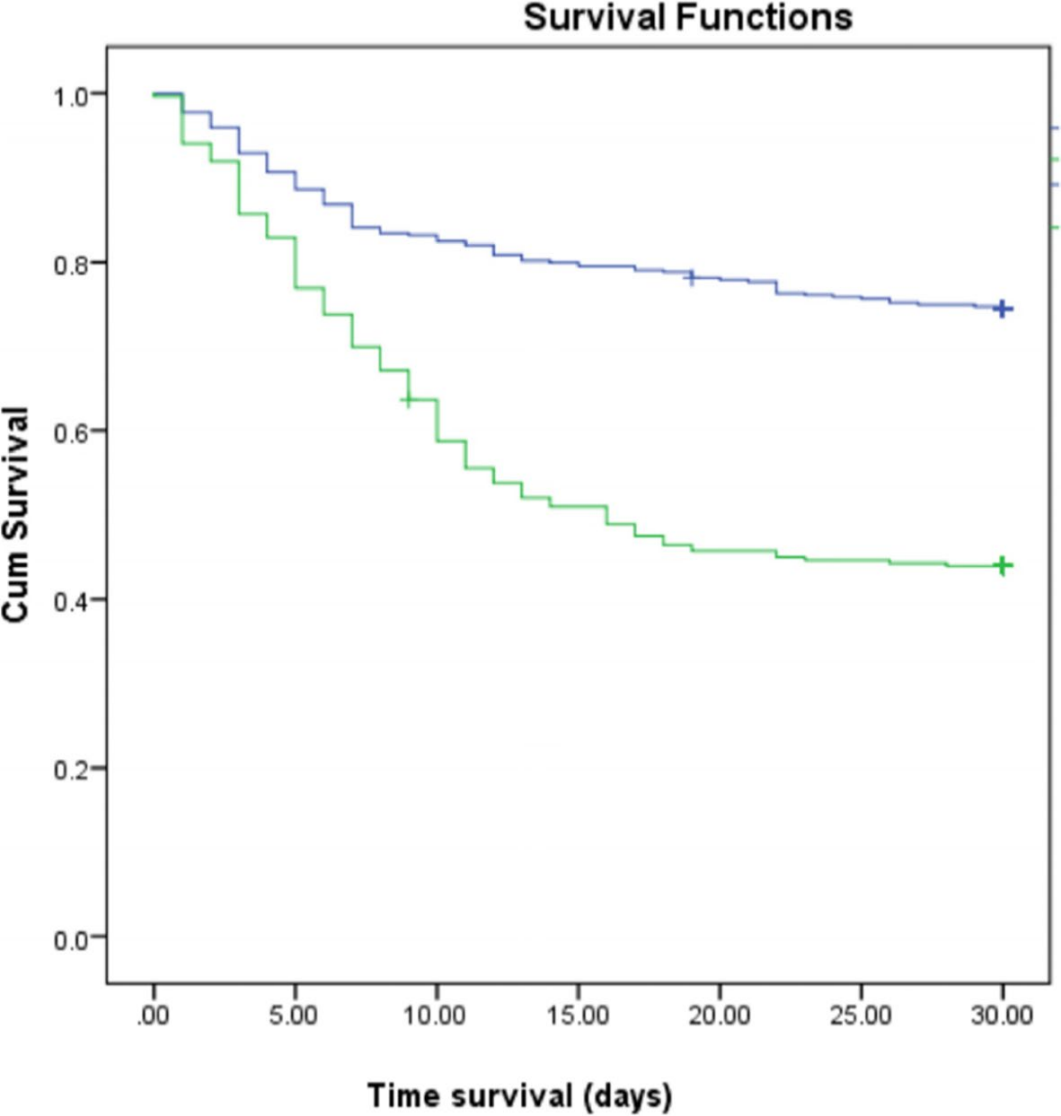
COVID-19 status. Blueline is Non COVID-19 and green line is COVID-19 (log-rank *p =* 0.001)

After conducting inferential analysis using Kaplan-Meier survival analysis (Fig. 2), we found that subjects with ten comorbidities had a more profound survival time than those without comorbidities (*p* < 0.05). The ten comorbidities were congestive heart failure (20.41 vs 22.5, *p =* 0.043), cerebrovascular disease (19.6 vs. 22.1, *p =* 0.008), dementia (14.4 vs. 22.1, *p =* 0.015), chronic pulmonary disease (18.1 vs. 22.0, *p =* 0.02), rheumatologic disease (7.0 vs. 21.9, *p =* 0.005), moderate to severe liver disease (16.1 vs. 22.1, *p =* 0.015), diabetes (19.6 vs. 22.5, *p =* 0.002), moderate-to-severe renal disease (17.3 vs. 22.6, *p =* 0.001), diabetes with chronic complications (15.3 vs. 22.1, *p =* 0.001), and hypertension (20.4 vs. 22.5, *p =* 0.005). On the other hand, subjects with COVID-19 also had a lower survival time than those without COVID-19 (*p* < 0.005) (Fig. 3).

## Discussion

Geriatric patients have a poorer rate of survival compared to younger people. Many factors contributed, including the changes in physiology and comorbidity. A study in 2007 showed that patients aged older than 80 years old had a higher mortality rate. Other studies have shown that the mortality of the elderly was high (20-55%) in the ICU [12, 13]. A multi-centre study showed that the length of stay of geriatric patients in ICU was between 10 to 23 days [14]. Based on our result, the mortality rate of geriatric patients in ICU was 38.6%, and the mean length of stay was 6.9 ± 7.0 days.

In the present study, we found high use of mechanical ventilation among geriatric ICU patients. The use of invasive mechanical ventilation was common not only in normal age but also in all geriatric patients admitted to the ICU. A study in a district hospital in Spain showed that about 39% of elderly patients in the ICU were intubated and required mechanical ventilation [15]. Another research in Glasgow also discovered that continuous assisted ventilation use contributed significantly to the risk of mortality in ICU patients. ICU patients with both non-invasive or invasive mechanical ventilation had a two times higher risk of death than those without the use [13]. A large data study in Taiwan revealed that 70% of intubated ICU patients were older than 65 years. Only 3 out of 10 elderly patients admitted to the ICU, both with acute respiratory failure and using mechanical ventilation, survived in one-year observation [16]. The mechanism causing this is diffuse alveolar damage. Several aetiologies such as cytokine-laden pneumonia, aspiration, direct injury by ventilation pressure may cause profound fluid and cellular exudation. The tissue overload then proceeds to hamper the perfusion of oxygen into the blood. Elderly patients had higher levels of inflammatory mediators and endothelial activation markers such as interleukins. Moreover, the aged population had a much higher Angiotensin-2 expression in circulation, meaning that SARS-Cov-2 was capable of infecting the bloodstream and causing cytokine storms in elderly patients [17].

Not only assisted ventilation, but our data also demonstrated a high use of vasopressors and inotropic agents among subjects. A similarly multi-centre study in France [14] revealed that the vasopressor requirement was quite high, with more than half of patients subjected to vasopressor infusion. The consistent use of vasoactive agents found in our research centres could occur due to sepsis, septic shock, or cardiogenic shock in elderly patients. In Indonesia, the most frequent type of vasopressor used is norepinephrine. With an average initial MSOFA score of 5 in our study, admitted patients to the ICU were in multiple organ failure states. On the other hand, a study in Libya [18] showed that inotropic use in the elderly in ICU was in one out of four patients and the mortality rate was nearly in two out of five elderly patients. Heart failure is understandably found in geriatrics, mainly because of a stiffening of arteries and a certain decreased ejection fraction of the heart due to ventricular muscle hypertrophy and fibrosis, especially in those with hypertensive heart disease. Although the use of inotropic was proven to be effective in acute settings, prolonged use was not recommended because of a high rate of documented mortality [19]. Dobutamine and dopamine, both potent adrenergic agonists, increased the ejection fraction of an already failed heart but also increase the risk of death in a chronic setting [19–21].

We found that several factors impact 30 days survival mortality rate in our study. It includes COVID-19 status, and several comorbidities, according to Charlson Index. One of the tools that classified the prognostic comorbidities and the comorbidities themselves is the Charlson index. We found that the higher the Charlson index, the higher the mortality rate. Score 5 had the highest mortality rate, reaching 2 out of 3 patients. A higher score indicated a poorer prognostic [22] Our result aligns with a previous study that patients with > 80 years old in ICU have poorer outcomes than in younger patients [23].

COVID-19 status has affected the survival rate in intensive care, especially in geriatrics. A study in Portugal discovered that the COVID-19 death rate was 16.8% among elderly patients admitted to the ICU. Furthermore, patients ≥70 years old have six times more likely to die than patients < 70 years old [6]. Even in short time follow-up, the mortality rate of geriatric patients with COVID-19 in ICU is relatively high, reaching up to 80% in several studies [24–26]. A comparison using Indonesian Task Force big data showed that although the most common age group admitted to the hospital in the COVID-19 pandemic was 31-45 years old, the elderly population experienced the most mortality rate (> 60 years old) with roughly 18% [27].

The high mortality rate in our geriatric patients is also related to pre-existing underlying diseases. Our study, which found ten particular comorbidities affecting the death rate, is in line with a previous study in Libya, which presented a significant association between mortality and comorbidities, such as diabetes, chronic pulmonary disease, asthma, malignant neoplasm, and immunosuppression patients [18]. A study in China [28] showed that the most common comorbidities found in the elderly were cerebrovascular diseases, diabetes, gastroduodenal ulcer, and tumour without metastasis. In Indonesia, the three most common comorbidities identified in all COVID-19 patients were vascular-related disorders, such as high blood pressure, diabetes, and cardiovascular disease [27]. Pre-existing diabetes mellitus is a characteristic found in more than 80% of fatal COVID-19 cases in patients older than 80 years [29]. A study in the US asserted that almost 90% of elderly and seriously ill patients with COVID-19 suffered from comorbidities such as hypertensive heart disease and diabetes mellitus [30]. As mentioned earlier and to conclude, multiple comorbidities increase the probability of mortality from COVID-19 [29]. Hypertension was the most frequent comorbid found in elderly patients in the ICU, followed closely by diabetes and moderate or severe renal disease. The activation of the renin-angiotensin-aldosterone system in several tissues influences arterial hypertension by constricting the vessels [31]. Conventional administration of antihypertensive agents, such as Angiotensin Receptor Blockers or Angiotensin-Converting Enzyme Inhibitors, positively alter the Angiotensin-Converting Enzyme 2 (ACE2) expression, making it easier for SARS-CoV-2 to infiltrate pneumocytes and ultimately deteriorating the severity and mortality of the infection. This condition, coupled with the stiffness of the artery wall, may worsen the disease progressivity due to increased systemic vascular resistance that then burdens an already increased myocardial demand [21, 31, 32].

In addition to mortality, the SOFA score was also used to determine the outcome of therapy. SOFA score consisted of physiological variables from respiratory, cardiovascular, liver, renal, and neurological systems. A low SOFA score in geriatric patients is taken into account for the survivability in COVID-19 [33]. However, in this study, SOFA scores on admission day and 30 days after ICU admission were not statistically different but clinically the result may be acceptable. We deduced the robust number of non-survived subjects 30 days after ICU admission; or because the length of stay was too long, both of which prompt the physiological function almost to return to baseline.

We found several limitations in our study. Firstly, there were obstacles in baseline day data collection. In this study, we frequently encountered either incomplete or missing records. Therefore, we may have missed more comorbidities or failed to calculate the precise SOFA score that accounted for the subjects and contributed to their condition before and on admission. Secondly, most hospitals we surveyed were central general public hospitals as we intentionally did not include private hospitals. Intensivists at those hospitals may have better resources and experience than intensivists in other less inferior or private hospitals, mainly because the public hospitals also act as teaching hospitals where they host residents and in-training consultants. Lastly, we admitted that there could also possibly be a distinguished disparity between urban and rural medical settings, thus our results may represent the standard of ICU care in bigger cities but not in lower-level hospitals. Future researchers should consider doing a more extensive and strictly monitored study with a greater sample size to minimize the aforementioned limitations.

## Conclusion

The findings of this study provided important information regarding comorbidities that are susceptible to the prognosis of geriatric patients receiving ICU care. In addition, the coronavirus 2019 undeniably exacerbates underlying comorbidities and delivers impact to the elderly patient, sequentially decreasing the survival rate in Indonesia.

## Data Availability

All data generated or analysed during this study are included in this published article.

## Abbreviations

AIDS: Acquired immunodeficiency syndrome
ASA: American Society of Anaesthesiology
COVID: Coronavirus disease
ICU: Intensive care unit
MMSE, MSOFA: Modified Sequential Organ Failure Assessment
PONV: Postoperative nausea and vomiting
KATI: The Indonesian College of Anaesthesiology and Intensive Therapy
PERDATIN: Indonesian Society of Anaesthesiologists and Intensive Therapy
